# A survey of castration methods and associated livestock management practices performed by bovine veterinarians in the United States

**DOI:** 10.1186/1746-6148-6-12

**Published:** 2010-03-03

**Authors:** Johann F Coetzee, Abbey L Nutsch, Laura A Barbur, Ryan M Bradburn

**Affiliations:** 1 Department of Clinical Sciences, College of Veterinary Medicine, Kansas State University, Manhattan, KS 66506-5601, USA; 2 Department of Diagnostic Medicine and Pathobiology, College of Veterinary Medicine, Kansas State University, Manhattan, KS 66506-5601, USA

## Abstract

**Background:**

Castration of male calves destined for beef production is a common management practice performed in the United States amounting to approximately 15 million procedures per year. Societal concern about the moral and ethical treatment of animals is increasing. Therefore, production agriculture is faced with the challenge of formulating animal welfare policies relating to routine management practices such as castration. To enable the livestock industry to effectively respond to these challenges there is a need for more data on management practices that are commonly used in cattle production systems. The objective of this survey was to describe castration methods, adverse events and husbandry procedures performed by U.S. veterinarians at the time of castration. Invitations to participate in the survey were sent to email addresses of 1,669 members of the American Association of Bovine Practitioners and 303 members of the Academy of Veterinary Consultants.

**Results:**

After partially completed surveys and missing data were omitted, 189 responses were included in the analysis. Surgical castration with a scalpel followed by testicular removal by twisting (calves <90 kg) or an emasculator (calves >90 kg) was the most common method of castration used. The potential risk of injury to the operator, size of the calf, handling facilities and experience with the technique were the most important considerations used to determine the method of castration used. Swelling, stiffness and increased lying time were the most prevalent adverse events observed following castration. One in five practitioners report using an analgesic or local anesthetic at the time of castration. Approximately 90% of respondents indicated that they vaccinate and dehorn calves at the time of castration. Over half the respondents use disinfectants, prophylactic antimicrobials and tetanus toxoid to reduce complications following castration.

**Conclusions:**

The results of this survey describe current methods of castration and associated management practices employed by bovine veterinarians in the U.S. Such data are needed to guide future animal well-being research, the outcomes of which can be used to develop industry-relevant welfare guidelines.

## Background

Castration of male calves destined for beef production is one of the most common livestock management practices performed in the United States amounting to approximately 15 million procedures per year [1]. Methods of castration are typically associated with physical, chemical or hormonal damage to the testicles [2]. In most production settings, physical castration methods are the most common. These can be subdivided into procedures involving surgical removal of the testes, or methods that irreparably damage the testicles by interruption of the blood supply using a castration clamp (Burdizzo castration), rubber ring or latex band [3]. Data describing the prevalence of each of these castration methods as performed by veterinarians in the United States are deficient in the published literature.

Benefits of castration include a reduction in aggression and mounting behavior of males causing fewer injuries in confinement operations and reduced dark-cutting beef [4,5]. Steers also have higher quality meat with increased tenderness and marbling. Carcasses from steers therefore command higher prices at market when compared with bulls [3]. Castration also prevents physically or genetically inferior males from reproducing and prevents pregnancy in commingled pubescent groups [2]. Although the benefits of castration are widely accepted in most countries, all methods of castration have been demonstrated to produce physiological, neuroendocrine, and behavioral changes indicative of pain and distress [2,6-9].

Societal concern about the moral and ethical treatment of animals is becoming more prevalent [10]. In particular, negative public perception of castration and dehorning is increasing, with calls for the development of practices to relieve pain and suffering in livestock [11]. Production agriculture is faced with the challenge of formulating animal welfare policies relating to routine management practices such as castration. To enable the livestock industry to effectively respond to these challenges there is a need for more data on management practices that are commonly being used in production settings [12]. The objective of this survey was to describe castration methods, adverse events and husbandry procedures performed by U.S. veterinarians at the time of castration. Such data are needed to guide future animal well-being research so that the outcomes can be used to formulate industry-relevant welfare guidelines.

## Results

Of the list of 1,972 email addresses to which this survey was sent, 189 U.S. veterinarians completed all the questions. This represents a crude response rate of 9.6 percent. The average time to complete the survey was 32 minutes. Thirty-one (1.5%) participants that started the survey did not complete all the questions and these data were excluded from the analysis.

### Demographic information

**Figure 1:**
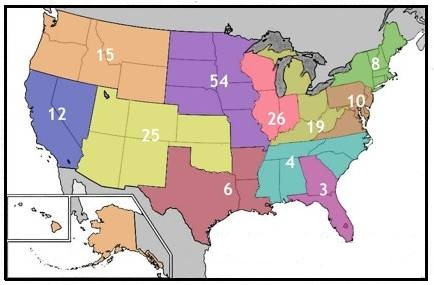
**Geographic location of respondents in the United States based on AVMA geographic district**.

Veterinarians responding to the survey were grouped according to American Veterinary Medical Association (AVMA) geographic district. The majority of respondents (54; 29%) were from District 7 (Iowa, Minnesota, Missouri, Nebraska, North Dakota, and South Dakota). The second highest participation (26; 14%) came from District 6 (Illinois, Indiana and Wisconsin) and District 9 (Arizona, Colorado, Kansas, New Mexico, Oklahoma, and Utah) (Figure 1).

**Figure 2:**
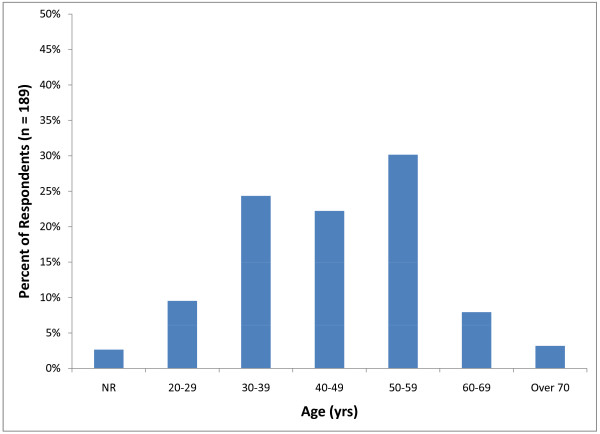
**Distribution of the percent of respondents in each age category (NR = not reported)**.

**Figure 3:**
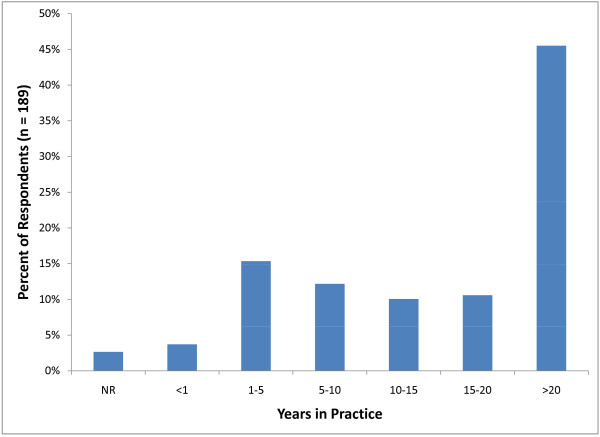
**Distribution of the percent of survey respondents by the number of years in practice (NR = not reported)**.

Respondents were most frequently in the age range of 50-59 years (57; 30%) followed by 30-39 years (46; 24%) (Figure 2). The majority of veterinarians participating in the survey (86; 45.5%) had been in practice for over 20 years. The second largest group of participants (29; 15%) consisted of relatively recent graduates (1-5 years) (Figure 3). Male veterinarians (146) accounted for 77 percent of those that completed the survey. Graduates from Iowa State University (27; 14%), Kansas State University (25; 13%) and The Ohio State University (15; 8%) accounted for over 30 percent of respondents. The majority of the practices represented in this survey comprised one (53; 28%) or two (32; 17%) veterinarians.

Twenty-seven percent of respondents (51) indicated that 11-20% of their gross practice income was derived from beef cattle. Seventy-two respondents (38%) indicated that the average size of beef breeding herds in their practice were between 1 and 49 head with 62 (33%) indicating that average herd size was between 100 and 499 head. Most respondents indicated that they were either not engaged in beef backgrounder operations (50; 26%) or they were involved in backgrounder operations of 100 - 499 head. Most respondents surveyed (117; 62%) were engaged in some aspect of beef feedlot operations. Six respondents indicated that 91 - 100% of their gross practice income was derived from beef cattle.

Similarly, 51 respondents (27%) indicated that 11-20% of their gross practice income was derived from dairy operations. Seventy-one respondents (37%) indicated that the average size of dairy herds in their practice were between 100 and 499 head. Most respondents (70; 37%) were not engaged in custom dairy calf rearing operations. Eight respondents indicated that 91 - 100% of their gross practice income was derived from dairy cattle.

### Castration methods

**Figure 4:**
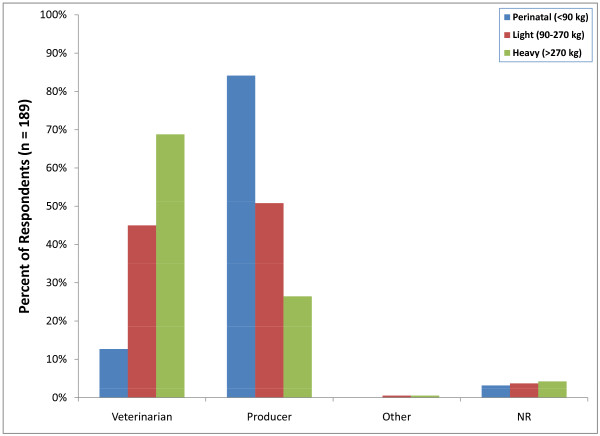
**Percent of respondents indicating who typically perform bovine castration (NR = not reported)**.

Over 83 percent (157/189) of respondents indicated that in their practices, producers were primarily responsible for performing castrations in perinatal calves less than 90 kg. In contrast, 129 respondents (68%) reported that castration of calves weighing more than 270 kg was conducted by a veterinarian. There were no reports of castrations performed by veterinary technicians (Figure 4).

**Figure 5:**
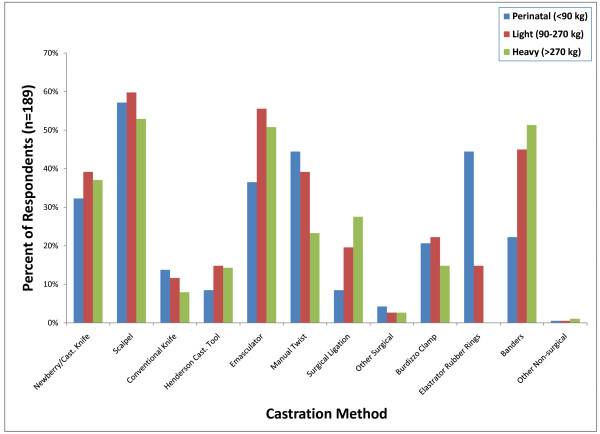
**Percent of respondents that indicated they perform each of the listed castration methods regardless of frequency**.

Surgical castration with a scalpel (108; 57%) followed by testicular removal by either manually twisting the testicles (84; 44%) or use of an emasculator (69/189; 36%) was the most frequently used method (Figure 5). Other methods of surgical castration that were used less often included using a Newberry Knife (61; 32%) or a conventional knife (26; 14%) to incise the scrotum and a Henderson Castration Tool (16; 8%) or surgical ligation (8%) to remove the testicles. Elastrator rubber rings (84; 44%) were the most commonly used non-surgical castration method employed in calves less than 90 kg. This was followed by banders (42; 22%) and the burdizzo clamp (39; 21%).

**Figure 6:**
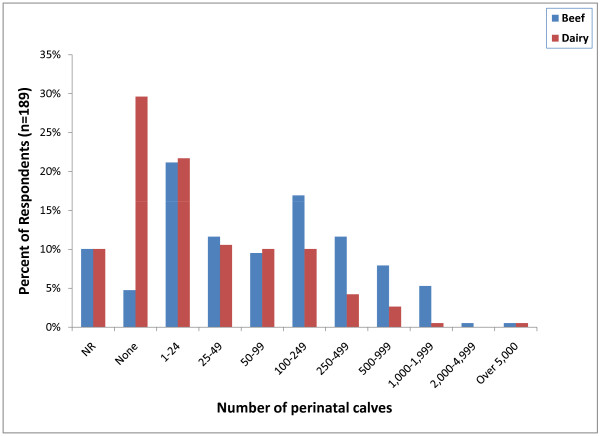
**Percent of respondents that indicated the approximate number of castrations in perinatal calves (<90 kg) performed per year (NR = not reported)**.

**Figure 7:**
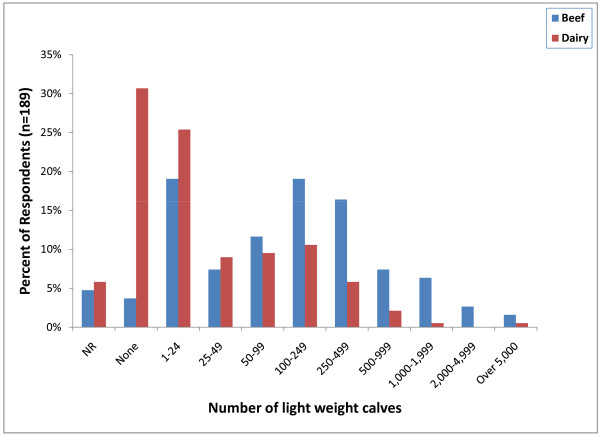
**Percent of respondents that indicated the approximate number of castrations in light weight calves (90-270 kg) performed per year (NR = not reported)**.

Approximately 90% of respondents (171) indicated that they do castrate some perinatal calves (calves weighing less than 90 kg). However, the actual number of calves castrated was relatively small with 40 respondents (21%) indicating that they only castrate between 1 and 24 perinatal beef calves per year and 56 (29%) indicating that they do not castrate any perinatal dairy calves (Figure 6). Ninety-five percent of respondents (180) indicated that they castrate light weight calves (90 - 270 kg). Thirty-six respondents (19%) only castrate between 1 and 24 light weight beef calves per year and the same number indicated that they castrate between 100 and 249 light weight calves per year. Fifty-eight respondents (31%) indicated that they do not castrate any light weight dairy calves and 48 (25%) only castrate between 1 and 24 light weight dairy calves per year (Figure 7). Surgical castration with a scalpel (113; 59%) followed by testicular removal with an emasculator (105; 55%) was the most common castration method used in light weight calves (Figure 6). Other methods of surgical castration that were applied less frequently included the use of a Newberry Knife (74; 39%), manual twisting to remove the testicles (74; 39%), surgical ligation of the testicles (37; 19%), the Henderson Castration Tool (28; 15%) and a conventional knife (22; 12%). Banders (85; 45%) were the most commonly used non-surgical castration method in light weight calves followed by the Burdizzo clamp (42; 22%) and elastrator rubber rings (28; 15%).

**Figure 8:**
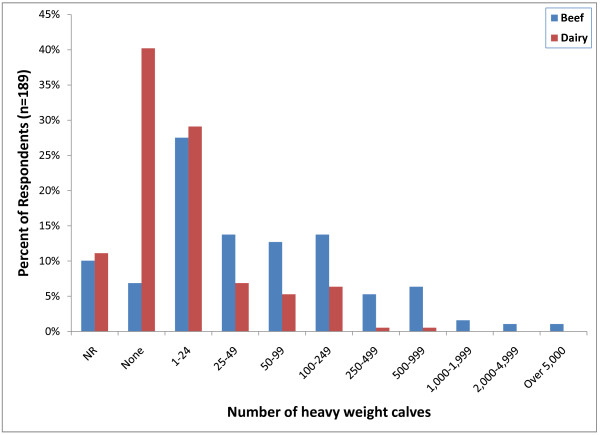
**Percent of respondents that indicated the approximate number of castrations in heavy weight calves (>270 kg) performed per year (NR = not reported)**.

Approximately 89% of respondents (169) indicated that they castrate heavy weight calves (>270 kg). Fifty-two respondents (27%) only castrate between 1 and 24 heavy weight beef calves per year, however 26 respondents (14%) indicated that they castrate between 100 and 249 heavy weight calves per year. Seventy-six respondents (40%) indicated that they do not castrate any heavy weight dairy calves and 55 (29%) only castrate between 1 and 24 heavy weight dairy calves per year (Figure 8). Surgical castration with a scalpel (100; 53%) followed by testicular removal with an emasculator (96; 50%) was also the most common castration method used (Figure 6). Other methods of surgical castration used included manual twisting to remove the testicles (44; 23%), the use of a Newberry Knife (70; 37%), surgical ligation (52; 28%), a Henderson Castration Tool (27; 14%) and a conventional knife (15; 8%). Banders (97; 51%) were also the most commonly used non-surgical castration method used in heavy weight calves followed by the Burdizzo clamp (28; 15%).

### Factors affecting selection of a castration method

**Figure 9:**
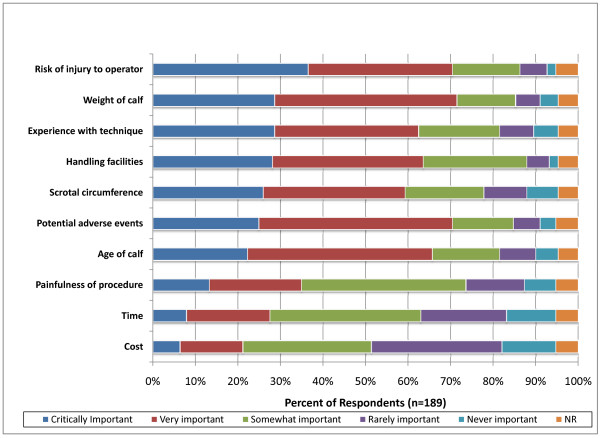
**Percent of respondents that indicated the relative importance of factors used to select a castration method in calves (NR = not reported)**.

For the majority of respondents (70; 37%), risk of injury to the operator was the most critically important consideration in the selection of castration method with 65 respondents (34%) indicating that this was very important (Figure 9). The weight of the calf was considered critically important by 54 respondents (28%) and very important by 82 respondents (43%). Experience with the castration technique was considered critically important by 56 respondents (29%) and very important by 64 respondents (34%). Other considerations, in order of critical importance to the participants, were handling facilities (54; 28%) scrotal circumference (49; 26%), adverse effects (48; 25%), age of calf (42; 22%), painfulness of procedure (26; 13%), time taken to conduct the procedure (15; 8%), and the cost of performing the procedure (12; 6%).

### Adverse events associated with castration

**Figure 10:**
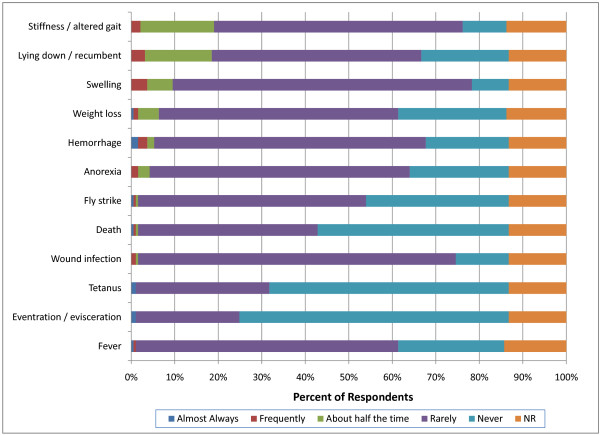
**Percent of respondents that indicated the frequency of adverse events associated with surgical castration in perinatal (<90 kg) calves (NR = not reported)**.

**Figure 11:**
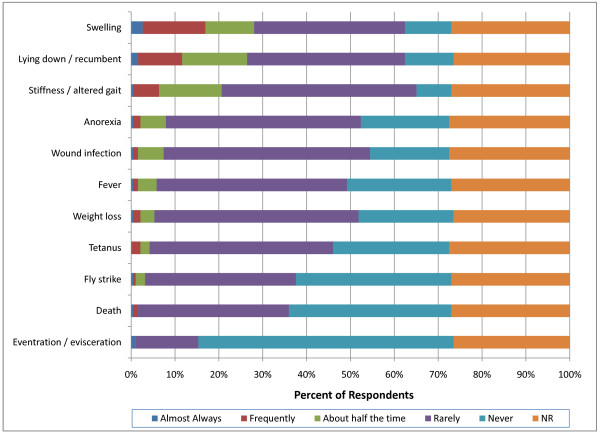
**Percent of respondents that indicated the frequency of adverse events associated with non-surgical castration in perinatal (<90 kg) calves (NR = not reported)**.

In perinatal calves (calves weighing less than 90 kg), 34 respondents (18%) indicated that stiffness/altered gait and recumbency was observed more than half the time following surgical castration (Figure 10). In contrast, 53 respondents (28%) indicated that swelling was observed more than half the time following non-surgical castration (Figure 11). This was significantly more than was reported for surgical castration (p = 0.0023). Furthermore, 50 respondents (26%) indicated that they observed recumbency greater than half the time following non-surgical castration which was significantly more than was reported for surgical castration (35; 18%) (p = 0.0002). However, these results should be interpreted with caution because respondents indicated that the majority of non-surgical castrations in the perinatal calves were performed by producers.

**Figure 12:**
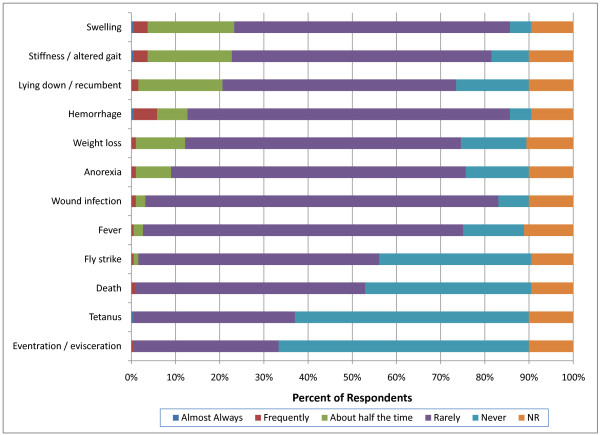
**Percent of respondents that indicated the frequency of adverse events associated with surgical castration in light weight (90-270 kg) calves (NR = not reported)**.

**Figure 13:**
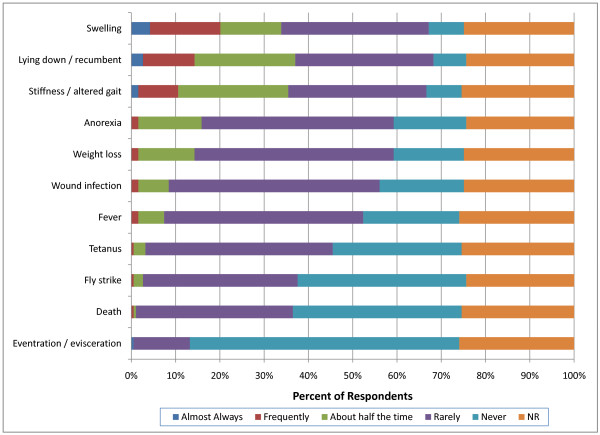
**Percent of respondents that indicated the frequency of adverse events associated with non-surgical castration in light weight (90-270 kg) calves (NR = not reported)**.

More respondents indicated that swelling, stiffness and recumbency occurred greater than half the time following both surgical and non-surgical castration in light weight calves (90-270 kg) compared with perinatal calves (p < 0.0001) (Figure 12). Furthermore, significantly more respondents reported hemorrhage more than half the time in light weight calves (14; 7%) following surgical castration compared with perinatal calves (6; 3%) (p < 0.0001). Comparison between non-surgical and surgical castration methods in light weight calves suggested that significantly more respondents believed that non-surgical methods produced swelling (45; 24% compared with 31; 16%); recumbency (47; 25% compared with 28; 15%); stiffness (44; 23% compared with 28; 15%) and anorexia (19; 10% compared with 12; 6%) (p < 0.0001) over half the time compared with surgical methods (Figure 13). Participants also associated wound infection more frequently with non-surgical castration (p = 0.02).

**Figure 14:**
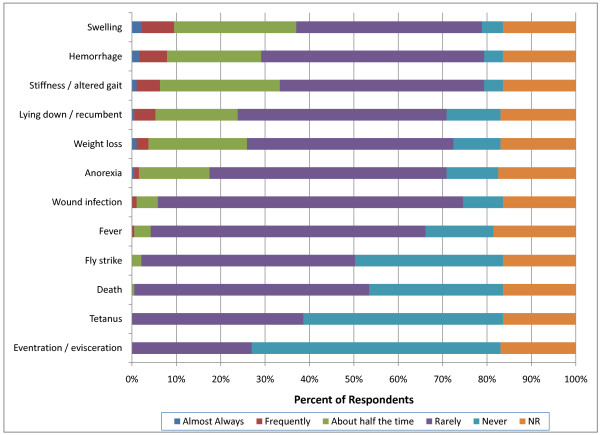
**Percent of respondents that indicated the frequency of adverse events associated with surgical castration in heavy weight (>270 kg) calves (NR = not reported)**.

**Figure 15:**
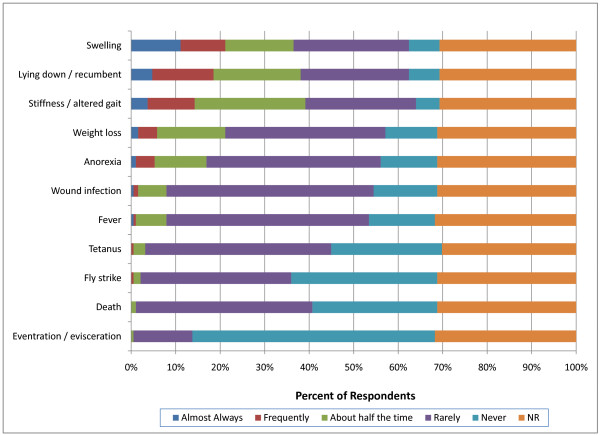
**Percent of respondents that indicated the frequency of adverse events associated with non-surgical castration in heavy weight (>270 kg) calves (NR = not reported)**.

More respondents indicated that swelling, stiffness and recumbency occurred more than half the time following both surgical and non-surgical castration in heavy weight calves (>270 kg) compared with light weight calves (p < 0.0001). As was the case with perinatal and light weight calves, veterinarians reported that non-surgical led to more frequent swelling, recumbency and stiffness in heavier calves (>270 kg) than surgical castration (p < 0.0001) (Figures 14 and 15). Hemorrhage was again more commonly observed following surgical castration in this weight class. Respondents also associated anorexia more with non-surgical castration rather than surgical castration.

### Ancillary management practices performed at the time of castration

**Figure 16:**
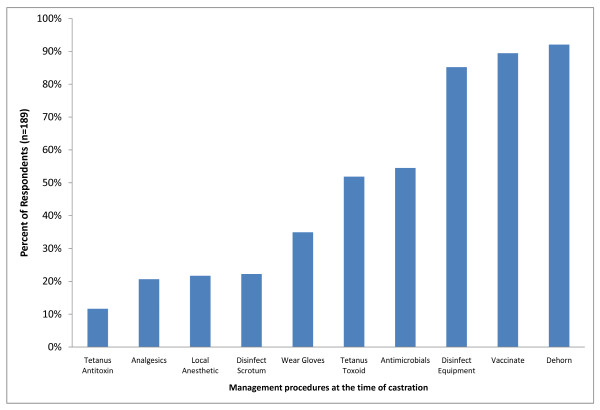
**Percent of respondents that indicated they perform other management practices at the time of castration**.

The results of the responses to questions about surgical practices, disease prevention, pain management and ancillary husbandry practices performed at the time of castration are summarized in Figure 16.

### Surgical practices

Sixty-two percent (118) of respondents indicated that they did not wear surgical gloves during castration with 19% (35) indicating that they only wear gloves during surgical castration. Seventy-seven percent of respondents (142) indicated that they do not routinely disinfect the scrotum prior to castration with 23% (42) indicating that they only disinfect the scrotum prior to surgical castration. Chlorhexidine (29; 15%) and iodine (25; 13%) were the most commonly used disinfectants prior to castration. Eighty-five percent of respondents (161) indicated that they routinely disinfect equipment between calves. Sixty percent (111) indicated that equipment was only disinfected after surgical castration with the remaining 25% (50) indicating that they disinfect equipment between animals after both surgical and non-surgical castration.

### Tetanus prophylaxis

Fifty-two percent of respondents (98) indicate that they routinely used tetanus toxoid injection at the time of castration; 4% (7) indicated that they only provide prophylaxis prior to surgical castration, 34% (65) prior to non-surgical castration and 14% (26) prior to both surgical and non-surgical castration. Only 28% of respondents (52) routinely administered tetanus toxoid to perinatal calves, 48% (90) to light weight calves and 54% (102) to heavy weight calves. Only 12% of respondents (22) routinely used tetanus antitoxin at the time of castration with 6% (12) indicating that this was for non-surgical procedures only. Only 9% of the total respondents (16) routinely administered tetanus toxoid to perinatal calves, 11% (20) to light weight calves and 15% (28) to heavy weight calves.

### Antimicrobial administration

Fifty-four percent of respondents (103) indicated that they routinely administer antimicrobials at the time of castration. Of these, 43% (82) indicated that they provide antimicrobial prophylaxis only prior to surgical castration with 11% (21) administering antimicrobials prior to both surgical and non-surgical castration. Sub-classification revealed that 14% (27) did not administer antimicrobials prior to perinatal calf castration and 28 respondents (15%) administered antimicrobials to only 1--10% of perinatal calves. In contrast, 34 respondents (18%) administered antimicrobial prophylaxis routinely to 91 - 100% of light weight calves with 34 respondents (18%) routinely administering antimicrobials to 91 - 100% of castrated heavy weight calves. Beta-lactams were the most common antimicrobial class administered prophylactically by 87 respondents (46%) followed by tetracyclines (42; 22%), macrolides (7; 4%) and florfenicol (6; 3%).

### Local anesthetic use

Twenty-two percent of respondents (42) indicated that they routinely administer local anesthetics (eg. lidocaine) prior to castration. Eighty-three percent of these (35/42) provided local anesthesia prior to surgical castration only with the remaining 17% (7/42) administering local anesthesia before surgical and non-surgical castration. Fifty-seven percent (24/42) that indicated they provide local anesthesia did not administer it to perinatal calves. Twenty-four percent of these (10/42) indicated that they either did not provide local anesthesia or they did only to 1-10% of light weight calves with 14% (6/42) indicating that they routinely administered local anesthetics to 91-100% of light weight calves. In contrast, 26% of respondents that administered local anesthetics prior to castration (11/42) did so in 91-100% of castrated heavy weight calves.

Thirty-eight percent of respondents (16/42) that used local anesthesia prior to castration administered a total volume of 5-10 ml of lidocaine with 38% (16/42) using 2-5 ml and 9% (4/42) using >10 ml. Sixty-four percent of respondents (27/42) indicated that they allowed 0-5 minutes to elapse between lidocaine administration and castration with the remaining 36% indicating that they waited 5-10 minutes for anesthesia to take effect.

### Systemic analgesic use

Twenty-one percent of respondents (40) indicated that they administer systemic analgesics at the time of castration. Forty-five percent of these (18/40) administered analgesia after surgical castration only, with the remaining 55% (22/40) following both surgical and non-surgical castration. Thirty-three percent of respondents (13/40) that used analgesics indicated that they did not administer these to perinatal calves with 35% (14/40) indicating that they used analgesics in only 1-10% of cases. In contrast, 43% of respondents (17/40) that use analgesics administered these to 1-10% of light weight calves with 13% (5/40) indicating that they routinely administered analgesics to 91-100% of cases. Similarly, 30% of respondents (12/40) that use analgesics administered these to 1-10% of heavy weight calves with 28% (11/40) indicating that they routinely administered analgesics to 91-100% of cases. Flunixin meglumine was the most common systemic analgesic administered by 38 respondents (95%). This was followed by alpha-2 agonists (13; 33%), opioids (4; 10%) and aspirin (4; 10%).

### Vaccination

Ninety percent of respondents (171) indicated that they vaccinate cattle at the time of castration. Clostridial vaccines were the most commonly administered vaccines (140/171; 82%) to perinatal calves followed by modified live multivalent viral vaccines against bovine respiratory disease (BRD) pathogens (99/171; 58%). A smaller number of these respondents indicated that they also vaccinate against *Mannheimia haemolytica *and *Histophilus somni *(33/171; 19%) and use killed multivalent viral vaccines against BRD (32/171; 19%) in perinatal calves. Similarly, in light weight calves, clostridial vaccines (156/171; 91%), modified live viral BRD vaccines (138/171; 81%), *Mannheimia haemolytica *and *Histophilus somni *vaccines (64/171; 37%) and killed viral BRD vaccines (41/171; 24%) were the most commonly used. This was also the case in heavy weight calves with clostridial vaccines (139/171; 81%) and modified live viral BRD vaccines (130/171; 76%) being the most prevalent.

### Dehorning practices

**Figure 17:**
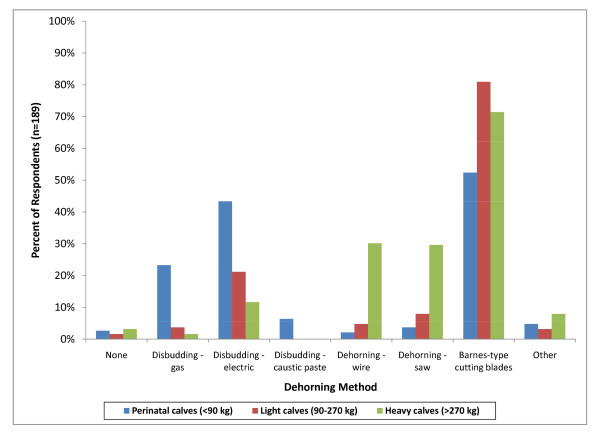
**Percent of respondents that indicated they perform each of the listed dehorning methods regardless of frequency**.

Ninety-two percent of respondents (176) indicated that they dehorn calves at the time of castration. In perinatal calves, horn removal with cutting blades (Barnes dehorner) (99; 52%), disbudding with an electric disbudding device (83; 43%), gas disbudding (44; 23%) and caustic paste disbudding (12; 6%) were the most common dehorning methods used (Figure 17). In light weight (153; 81%) and heavy weight (135; 71%) horn removal with cutting blades (Barnes dehorner) were the most common dehorning methods.

### Other management practices

In perinatal calves, weaning (8; 4%), hormone implanting (66; 35%), tagging (97; 51%), freeze branding (4; 2%) and hot iron branding (46; 24%) were also performed at the time of castration (Figure 16). These procedures were performed more frequently in older, light weight calves with weaning (60; 32%), hormone implanting (104; 35%), ear tagging (92; 48%), freeze branding (5; 2%) and hot iron branding (39; 21%) reportedly also performed at the time of castration. This is similar to the situation in heavy weight calves where weaning (57; 30%), hormone implanting (99; 52%), tagging (82; 43%), freeze branding (5; 2%) and hot iron branding (33; 17%) were also performed at the time of castration.

### Additional comments

Nine respondents provided additional comments indicating that producers were primarily involved in castrating calves in their practice areas. Four respondents indicated that the absence of FDA-approved analgesic compounds and the cost associated with analgesia was an impediment to widespread use at the time of castration. These respondents also indicated that producers were resistant to incurring the additional cost associated with providing analgesia routinely at castration. One respondent indicated that the survey was too long.

## Discussion

Approximately 15 million calves are castrated in the United States annually [1] yet little has been published with regards to methods of castration used by U.S. veterinarians. The objective of this survey was to describe castration methods, adverse events and husbandry procedures performed by bovine practitioners at the time of castration. The results of this survey suggest that surgical castration with a scalpel followed by testicular removal using either manual twisting (cattle <90 kg) or an emasculator (cattle >90 kg) are the most common castration methods used by veterinarians. Survey participants indicated that the potential risk of injury to the operator, the size of the animal, the availability and quality of handling facilities and experience with the technique were the most important factors considered when choosing a castration method. The most prevalent adverse events observed by respondents following castration were swelling, stiffness and increased lying time. In terms of preoperative analgesia, one in five respondents reported providing systemic analgesia or local anesthetic at the time of castration. It is noteworthy that approximately 90% of respondents report that they vaccinate and dehorning calves at the same time as these are castrated. Over half the respondents reported using disinfectants, prophylactic antimicrobials and tetanus toxoid in an attempt to reduce complications associated with castration. To our knowledge, this is the first report describing bovine castration methods and associated management practices employed by veterinarians in the United States. These data are necessary to guide future animal well-being research so that the outcomes can be used to develop industry-relevant welfare guidelines.

Some caution is appropriate in interpreting the results of the present study given the relatively low crude response rate of 9.6% which may have introduced a degree of non-response error. However, it is relevant that a recent survey of the same population of rural veterinarians conducted by the Food Supply Veterinary Medicine Coalition had a 10.4% response rate (133/1,280) in new graduates and an 8% response rate in long term veterinarians [13]. This suggests that low response rates may be typical of this study population in the United States. Several recent studies have also challenged the presumption that lower survey response rates imply lower survey accuracy [14]. This suggests that the results presented provide a meaningful first attempt at describing castration methods used by veterinarians in the U.S.

Other potential sources of bias in this study are similar to those highlighted in previous surveys and include coverage bias, sampling error, measurement error and response bias [15]. In terms of addressing coverage bias and sampling error, all email recipients of the invitation to participate in the survey identified themselves as veterinarians. However, it is significant that the mailing list used did not differentiate between AABP or AVC members in industry, practice and academia. The current AABP membership list only identifies 66% of members as being associated with veterinary practices. Therefore, not all survey recipients were involved in castrating calves but all were included in the denominator of the response rate calculation. This may have contributed to the lower response rate. Nonresponse could also be attributed to the length of the survey, the time of the year the survey was conducted (September - November) and that this same population had been recently surveyed for analgesic drug use [16] and job satisfaction [13]. Measurement error was reduced by pretesting the survey on veterinarians associated with the Beef Cattle Institute at Kansas State University and the AABP-AWC. In common with previous reports, response bias ("faking good" biases) was not assessed in this survey [15]. However, the effect or response bias was minimized by providing assurances that all data were confidential and that the researchers were not looking for right or wrong answers.

The demographic characteristics of the respondents in this survey were similar to those participating in a recent Canadian survey [15]. Both surveys enrolled more men who were older and engaged in smaller practices than have been reported in companion animal surveys. The majority of respondents from this study were from Iowa, Minnesota, Missouri, Nebraska, North Dakota and South Dakota which are states that account for 24% of the U.S. cattle inventory [1]. The second highest participation came from Illinois, Indiana and Wisconsin and Arizona, Colorado, Kansas, New Mexico, Oklahoma, and Utah which account for a further 23% of the U.S. cattle inventory [1].

Surveys of methods used by veterinarians to castrate calves are deficient in the published literature. However, results from similar surveys of farmers in the United Kingdom, New Zealand and the United States have been reported [17-19]. Since these surveys involve distinctly different populations, direct comparison between these reports and the present study should be interpreted with caution. However, in the absence of analogous studies, some comparisons between these reports may be relevant. Kent and others found that non-surgical castration methods were preferred by British farmers with 43% of respondents indicating that they used Burdizzo clamp castration [17]. However, UK producers preferred surgical castration in older calves with 68% indicating that these were conducted by veterinarians. These results are in agreement with the results presented here with 68% of respondents indicating that veterinarians were primarily involved in castrating heavy weight calves with 58% indicating that they prefer surgical castration methods. The preference for U.S. veterinarians to perform surgical castrations is likely due to client expectations and a greater level of comfort and experience with surgical procedures.

Removal of testicles with a blade was the most common method of castration performed by almost 50% of beef producers in the United States as reported by the NAHMS survey [19]. Only 3.5% of producers report using a burdizzo clamp compared with 39.5% of producers that report using a rubber ring or tubing in calves less than 3 months of age. Stafford and others reported that 85% of New Zealand producers preferred rubber ring castration, especially in the first 3 months of life, with 18% performing surgical castration mostly in older calves [18]. In contrast with the U.K survey, only 2.7% of New Zealand producers indicated that they used a veterinarian to perform castrations. This difference is likely due to the legislative requirement in the UK for older calves to be castrated by a veterinarian [20]. It is noteworthy that banding of heavy weight calves was not reported in the British and New Zealand producer survey yet over 53% of U.S. veterinarians report using banders to castrate heavy weight calves. Furthermore, neither survey reported the use of the Henderson castration tool to castrate calves. This finding likely represents differences in regional preferences between the study locations.

Factors affecting selection of a castration method have not been described in the published literature. For the majority of respondents the risk of injury to the operator was the most critically important consideration in the selection of castration method. This would seem to contradict the finding of our survey that surgical castration, which is arguably more hazardous than non-surgical castration, is preferred by veterinarians. However, as stated previously, the preference for using surgical castration methods by veterinarians is likely due to client expectations that surgical as opposed to non-surgical castration is performed by veterinarians. Concern for operator safety is justified based on a recent survey of Australian veterinarians which found that cattle related injuries accounted for 22% of all the significant injuries reported by veterinarians [21]. Scalpel or knife injuries accounted for 28% of the surgical injuries reported. It is noteworthy that only 13% of veterinarians considered the painfulness of procedure to be a critically important consideration in the selection of castration method. This finding is in agreement with a recent Canadian survey where veterinarians gave castration up to 6 months of age the lowest pain rating (4.6/10) with dehorning in the same age group receiving a pain rating of 7.2/10 [15].

The incidence of complications associated with castration reported by British farmers was low with 28% indicating that complications were most commonly associated with surgical castration [17]. A few New Zealand respondents reported swelling (2.9%), deaths (1%), infection (0.7%) or bleeding (0.6%) particularly after surgical castration [18]. Neither survey related the incidence of complication to weight or age at the time of castration. In the present report, more veterinarians indicated that swelling, stiffness and recumbency occurred more than half the time following both surgical and non-surgical castration in heavy weight calves (>270 kg) compared with perinatal and light weight calves (p < 0.0001). In contrast with previous reports, U.S. respondents indicated that more complications were associated with non-surgical than surgical castration. Furthermore, the overall incidence of adverse events reported by veterinarians in the present survey was higher than previously reported by producers. This difference is likely due to veterinarians being specifically consulted about adverse events following castration and may thus be exposed to a biased population.

The effect of age and method of castration on the health and performance of calves has been reviewed elsewhere [2]. Several studies have compared the effects of surgical and non-surgical castration in bulls but results have been conflicting. Stafford and others found that peak cortisol response was higher in 2-4 month old calves after latex band castration compared with surgical castration but this difference was not statistically significant [22]. These researchers also found that cortisol response remained above baseline levels for 180 minutes following both surgical and non-surgical castration. Fisher and others concluded that banding produced fewer acute effects, but greater suppression of growth compared to surgical castration in 9 and 14 month old bulls [23]. Several other studies have also reported a more significant decrease in weight gain in banded calves compared with calves castrated surgically [24,25]. However, a recent Canadian study found that bulls castrated with a band had a lower occurrence of undifferentiated fever and improved average daily gain and carcass weight than bulls castrated surgically [26]. These conflicting research findings together with the results of this survey suggest that further research to compare the welfare implications of surgical and non-surgical castration methods and the optimal age of castration is needed.

In the British livestock producer survey, 90% of farmers attempted to control or prevent infection after surgical castration with 20% administering intramuscular antimicrobial prophylaxis [17]. Thirty-nine percent of respondents reported sterilizing equipment and 2% vaccinated against tetanus. In New Zealand 16.5% of producers boiled equipment, 8.4% washed the scrotum and 4.5% used antibiotics only with surgical castration [18]. Furthermore, only 20% of producers vaccinated calves at the time of castration. The results of the present study suggest that U.S. veterinarians are more likely to institute measures to control or prevent infection following castration than producers given the higher prevalence of disinfectant, antimicrobial and vaccine use.

Negative public perception of castration and dehorning is mounting, with increasing call for the development of practices to relieve pain and suffering in livestock [11]. Several studies have demonstrated that local anesthesia alone or combined with systemic analgesic drug administration prior to castration mitigates physiological, neuroendocrine, and behavioral changes usually associated with pain and distress [2,6-9,27]. In spite of this, only 10% of New Zealand producers report using local anesthesia in calves castrated surgically [18]. Although the use of local anesthetics at the time of castration is mandated in the United Kingdom [20], only 43% of British veterinarians were reported to use local anesthesia in calves older than 8 weeks of age [17]. A recent survey of Canadian veterinarians and their use of analgesics revealed that only 6.9% of beef calves and 18.7% of dairy calves ≤ 6 months old and approximately 20% of beef calves and 33% of dairy calves >6 months old received an analgesic at the time of castration [15]. The authors cite a lack of approved, long-acting, and cost-effective analgesics with established withdrawal times in Canada as one explanation for these findings. These results are in agreement with the findings of the present study where only 22% of respondents indicated that they routinely administer local anesthetics and only 21% indicated that they use systemic analgesics prior to castration.

Several organizations, including the National Cattlemen's Beef Association [28] and the American Veterinary Medical Association [3], have stated that pain and physiologic stress resulting from castration should be minimized. Available methods of minimizing pain and stress include application of local anesthetics and the administration of analgesics [2]. It is significant that there currently are no analgesic drugs approved for the alleviation of pain in livestock in the United States. The FDA Center for Veterinary Medicine guidance for the development of effectiveness data for NSAIDs indicates that validated methods of pain assessment must be used for a drug to be indicated for pain relief in the target species [29]. This requirement explains the lack of analgesic drugs approved for pain relief in livestock in the United States because there currently are no validated methods of pain assessment in food-producing animals.

A previous survey of U.S. dairy producers found that gas or electric dehorning irons were used on 67% of dairies with 67% of respondents indicating that calves were dehorned by 8 weeks of age [12]. In contrast, beef producers in the U.S. report using saws, Barnes or keystone guillotine dehorners in almost 40% of cases with the average age of dehorning reported to be around 120 days [19]. The results of the present survey indicate that Barnes cutting blade dehorners were preferred by veterinarians in all classes of cattle although 43% indicated that they performed disbudding with electric dehorning irons in perinatal calves. It is noteworthy that 92% of veterinarians surveyed in this report indicate that they also dehorn calves at the time of castration. This is in contrast with New Zealand producers where only 9.1% of respondents indicate that they disbud or dehorn calves at the time of castration [18]. Several studies have examined the behavioral, physiological and neuroendocrine effects of dehorning and castration conducted separately [2,3]. However, research into the cumulative effect of these procedures conducted concurrently is currently deficient in the published literature.

## Conclusions

The results of this survey describe current methods of castration and associated practices in the U.S. Our findings suggest that further research to compare the welfare implications of surgical and non-surgical castration methods and the optimal age of castration is needed. These results also indicate that routine analgesic drug administration at the time of castration is relatively uncommon in the U.S. although the data are consistent with survey results from other territories. Furthermore, this survey identifies the need for more research into the welfare implications of concurrent dehorning and castration and the effect of other management practices performed at the time of castration.

## Methods

### Survey design

Survey questions designed to identify current castration methods and associated management practices employed in cattle production systems in the United States were developed. The survey was constructed for electronic dissemination using survey software developed at Kansas State University (Axio Learning Systems, Manhattan, KS). A draft survey was pre-tested on veterinarians associated with the Beef Cattle Institute at Kansas State University (10 veterinarians) and the American Association of Bovine Practitioners Animal Welfare Committee (AABP-AWC) (30 veterinarians) to refine the structure and clarify areas of ambiguity. This survey was also examined by the Institutional Review Board (IRB) at Kansas State University and deemed exempt from full IRB review (KSU IRB #4406).

The survey was composed of 122 questions divided into 3 sections. Question types were structured similar to previous studies [30] to include percentages, yes/no answers and selection of the most appropriate answer from a list. Opportunities were also provided for respondents to provide additional feedback and to make general comments on the survey. A copy of the survey is available on request from the corresponding author.

The first section of the survey collected demographic information including age, gender, years in practice, education, location of the practice (by State), number of veterinarians in the practice, practice composition and species focus including estimates of average practice herd size. The second section surveyed methods of castration used by veterinarians. A series of follow-up questions queried complications associated with castration and the relative importance of a list of animal and management related factors in the selection of castration method. This section was subdivided into three parts by a lead question to determine if respondents were involved in castrating a particular weight class of calf. For the purpose of the survey the cattle population eligible for castration was subdivided into perinatal calves (0-200 lbs) (0-90 kg); light weight calves (200-600 lbs) (90-270 kg) and heavy weight calves (>600 lbs) (>270 kg). If respondents indicated that they were not involved in castrating one of these classes they were automatically directed to the next section of the survey.

For each weight class, respondents were asked to provide an estimate of the number of calves castrated annually and the percentage of these castrated using either surgical or non-surgical methods. Surgical castration options included a Newberry/Castration Knife, scalpel, conventional knife to incise the scrotum followed by testicular removal by manual twisting, surgical ligation, an emasculator or a Henderson Castration Tool. Non-surgical castration options included the burdizzo clamp, elastrator rubber rings, latex banders (eg. Callicrate^®^, Tri-band^® ^or California^® ^Banders) and other respondent-specified methods. The survey did not specifically differentiate between banders equipped with a tension gauge (Callicrate and Tri-band) and those that are not (California Bander).

In addition, respondents were asked how often (almost always; frequently; about half the time; rarely; never) they observed specified adverse events following either surgical or non-surgical castration methods. Specific adverse events surveyed included hemorrhage, swelling, wound infection, fly strike, fever, anorexia, weight loss, recumbency, stiffness, tetanus, eventration and death. Finally, the relative importance (critically important, very important, somewhat important, rarely important, never important) of a list of animal, operator and management system considerations in the respondents decision regarding which castration method to use was surveyed. Specific considerations that were listed included calf age, weight, scrotal circumference and operator experience level, risk of adverse events, perceived painfulness, handling facilities, cost, time and risk of injury to the operator.

The third section of the survey was designed to investigate ancillary management practices and husbandry procedures performed at the time of castration. These included questions about surgical practices, pain management and disease prevention. Questions about surgical practice included the use of surgical gloves, disinfection, tetanus vaccination and administration of antimicrobials. Pain management procedures included the use of local anesthetics and systemic analgesics. Other routine husbandry procedures performed at the time of castration included vaccination, dehorning, weaning, tagging, hormone implanting and branding.

### Survey protocol

The survey was conducted between 28 September 2007 and 14 November 2007. An invitation to participate in the survey was sent to email addresses of 1,669 members of the American Association of Bovine Practitioners (AABP) and 303 members of the Academy of Veterinary Consultants (AVC). All invited participants subscribed to either the AABP-L email discussion listserve AABP-L@listserv.umd.edu or the AVC-L discussion list AVC-L@listserv.unl.edu or in some cases both. An email invitation to participate in the survey explained the purpose of the study, provided assurance of confidentiality and sought permission for the anonymous responses to be published. The invitation also contained an embedded hyperlink to connect participants to the survey questions hosted on the Kansas State University server. Following initial circulation, reminders were posted to the lists on 20 October 2007 and 10 November 2007. The survey was closed to further participation 6 weeks after the initial offering.

### Statistical analysis

Data were entered into a spreadsheet (Microsoft Excel^® ^2003, Microsoft Corporation, Redmond, WA) where results from partially completed surveys were removed from the analysis.

Data describing demographic information, castration method by weight class, adverse events and management practices performed at the time of castration were generated using means and frequency tables. Data are presented as the number of participants in each response category and the percentage of the total number of survey participants (189). Hypothesis tests were conducted using JMP analytical software (SAS Institute, INC, Cary, NC, USA). Two-way comparisons were performed on selected parameters using 2 × 2 contingency tables with significance tested by chi-square or 2-tailed Fisher's exact test (for instances where there were fewer than 5 observations/cell) [30]. Statistical significance was designated *a priori *as a p-value less than or equal to 0.05.

## Authors' contributions

JFC conceived of the study, participated in its design and coordination, performed the statistical analysis and drafted the manuscript. ALN participated in the design of the study, and prepared the data for statistical analyses and helped draft the manuscript. LAB and RMB processed the survey results, cleaned the data and prepared the figures for publication. All authors read and approved the final manuscript.
